# The Prognostic Role of Neutrophil-to-Lymphocyte Ratio in Patients Hospitalized with Acute Pulmonary Embolism

**DOI:** 10.3390/jcm10184058

**Published:** 2021-09-08

**Authors:** Orly Efros, Tal Beit Halevi, Eshcar Meisel, Shelly Soffer, Noam Barda, Omri Cohen, Gili Kenet, Aharon Lubetsky

**Affiliations:** 1 National Hemophilia Center and Thrombosis & Hemostasis Institute, Sheba Medical Center, Ramat-Gan 5262000, Israel; omricmd@gmail.com (O.C.); Gili.Kenet@sheba.health.gov.il (G.K.); Aharon.Lubetsky@sheba.health.gov.il (A.L.); 2 Sackler Faculty of Medicine, Tel Aviv University, Tel Aviv 6927846, Israel; talbh142@gmail.com (T.B.H.); eshcarme@gmail.com (E.M.); 3 Department of Internal Medicine “D”, Sheba Medical Center, Ramat-Gan 5262000, Israel; 4 Assuta Medical Center, Ashdod 7747629, Israel; soffer.shelly@gmail.com; 5 Ben-Gurion University of the Negev, Be’er Sheva 8410501, Israel; 6 Clalit Research Institute, Clalit Health Services, Ramat-Gan 6578898, Israel; noambard@gmail.com; 7 Department of Biomedical Informatics, Harvard Medical School, Boston, MS 02115, USA; 8 Amalia Biron Research Institute of Thrombosis & Hemostasis, Sheba Medical Center, Ramat-Gan 5262000, Israel

**Keywords:** pulmonary embolism (PE), neutrophil-to-lymphocyte ratio (NLR), venous thromboembolism (VTE)

## Abstract

Early risk stratification is essential for determining the appropriate therapeutic management approach of pulmonary embolism (PE). This study aimed to evaluate the prognostic value of the neutrophil-to-lymphocyte ratio (NLR) in patients hospitalized with acute pulmonary embolism by investigating its association with mortality in a large-scale population diagnosed and hospitalized with acute PE. We retrieved all consecutive patients hospitalized in an internal medicine department or an intensive care unit in a tertiary medical center from December 2007 to April 2021 with a discharge diagnosis of pulmonary embolism. A total of 2072 patients were included. Patients with above-median NLR (i.e., 5.12) had a higher 30-day mortality risk (adjusted odds ratio (aOR), 2.82; 95% confidence interval (CI) 2.14–3.70) and higher one-year mortality risk (aOR, 2.51; 95% CI 2.04–3.08). Similar trends were demonstrated in a sub-analysis of patients without cancer and hemodynamically stable (i.e., systolic blood pressure over 90 mmHg). Furthermore, the median hospital length of stay in patients with an elevated NLR was higher, and so was the in-hospital mortality rate. Elevated NLR in acute PE is associated with a worse short-term and long-term prognosis and with a longer duration of hospitalization.

## 1. Introduction

Pulmonary embolism (PE) is the third most common cause of cardiovascular mortality worldwide [1]. There are approximately 200,000 annual hospital admissions for PE in the United States, causing a significant healthcare burden [2,3]. Deaths attributable to PE occur mainly within the first week following diagnosis, but related morbidity is seen years later [4,5]. Therefore, accurate early risk stratification is essential for determining the appropriate therapeutic management approach [6].

Inflammation has a key role in the pathogenesis of venous-thromboembolism [7,8,9,10]. Von Bruhl et al. demonstrated in vivo that the initiating stimulus for deep vein thrombosis (DVT) development is accounted for by the adherence of monocytes and neutrophils in the blood to endothelium [11]. Additionally, it was shown that neutrophils are essential for thrombus propagation [12].

The neutrophil-to-lymphocyte ratio (NLR) is a biomarker that reflects the balance between systemic inflammation and immunity [13]. It has been assessed as a prognostic biomarker in several diseases, including malignancies and cardiovascular diseases, and was demonstrated to be associated with the disease course and outcome [14,15,16]. In recent years, the NLR has also been proposed to have a prognostic role in acute PE [12,17]. However, previous research investigating NLR in acute PE has been limited to relatively small cohorts and restrictive inclusion criteria [18,19,20,21,22,23,24].

In this study, we aimed to assess the prognostic value of NLR by investigating its association with mortality in a large-scale population diagnosed and hospitalized with acute PE.

## 2. Methods

### 2.1. Study Design and Population

This is a retrospective cohort study. Data were obtained from electronic medical records (EMR) at the Sheba Medical Center, a large tertiary medical center operating in Israel. We retrieved all consecutive patients hospitalized with a discharge diagnosis of PE admitted to internal medicine departments or an intensive care unit (ICU) from December 2007 to April 2021. All patients included were 18 years old or older at the time of diagnosis and had at least one complete blood count in the 24 h following admission. If there was more than one complete blood count performed during that time, the first test was chosen. These study results are reported in accordance with the STROBE guidelines [25].

### 2.2. Data Collection and Outcomes

For each patient, baseline demographic characteristics and clinical information were extracted from the EMR. Clinical data included medical comorbidities, home medications, vital signs on admission (body temperature, systolic blood pressure, oxygen saturation), body mass index (BMI), and laboratory tests. Preexisting comorbidities were defined based on the International Classification of Disease, 10th Revision codes [26]. Comorbidities included prior PE and DVT, heart failure (HF), atrial fibrillation (AF), ischemic heart disease (IHD), chronic kidney disease (CKD), malignancy, chronic obstructive pulmonary disease (COPD), diabetes mellitus (DM), hypertension, cerebrovascular accident (CVA), and dyslipidemia. If death occurred outside the hospital, mortality dates were obtained from governmental mortality records.

The primary exposure was NLR, as calculated for each patient using the index blood count. For some of the analyses, NLR was categorized as elevated or not elevated using the median NLR from the sample as the threshold.

The two primary outcomes evaluated were patients’ mortality risk within 30 days and one year from admission. Secondary outcomes included in-hospital mortality and hospital length of stay.

### 2.3. Statistical Analysis

Baseline clinical data were compared between patients with elevated NLR and non-elevated NLR. Continuous variables were compared using the Mann–Whitney-Wilcoxon test, and categorical variables were compared using the Chi-square test.

Covariates for the multivariable models were pre-specified based on clinical relevance.

Adjusted odds ratios (OR) for 30-day and one-year mortality were estimated using logistic regression. The models were adjusted for age, sex, preexisting comorbidities (AF, IHD, HF, hypertension, DM, CKD, CVA, COPD, malignancy), and low systolic blood pressure at admission (defined as <90 mm of mercury (mmHg)). To test the persistence of this association in hemodynamically stable patients (i.e., systolic blood pressure of 90 mmHg and above) and non-cancer patients, we performed a sub-analysis in these sub-groups.

The crude association between elevated NLR and mortality was modeled using Kaplan–Meier curves and compared using the Log Rank test.

To better quantify the dose–response relationship between NLR and mortality, the exposure was further modeled as a continuous value using a thin-plate spline in a generalized additive model. The model was adjusted for the same variables as specified for the logistic regression model analysis.

Hospital length of stay and in-hospital mortality were compared between patients with elevated NLR and non-elevated NLR using the Mann–Whitney–Wilcoxon test and the Chi-square test, respectively.

Data analyses were performed using the R programming language (R Development Core Team, version 4.0.3, Vienna, Austria).

## 3. Results

From December 2007 to April 2021, a total of 2182 patients over the age of 18 were discharged with a primary diagnosis of PE from the internal medicine wards and the ICU at the Sheba Medical Center. Out of these 2182 patients, 110 were excluded as no CBC was available up to 24 h from admission.

Patients’ characteristics are presented in Table 1. The median NLR among the study population was 5.12. Comparing patients below and above median NLR showed that patients who had above-median NLR were older and had more underlying diseases. In addition, their pulse rate was higher, and their systolic blood pressure was lower at presentation (Table 1).

Elevated NLR had a crude OR of 3.72 (95% CI 2.87–4.83) and 3.1 (95% CI 2.56–3.75) for 30-day mortality and one-year mortality, respectively.

The adjusted ORs for 30-day mortality and one-year mortality are presented in Table 2. Adjustment for sex, age, low systolic blood pressure at presentation and comorbidities yielded elevated NLR as a risk factor for 30-day mortality (OR, 2.82; 95% confidence interval (CI) 2.14–3.70) and one-year mortality (OR, 2.51; 95% CI 2.04–3.08) in patients hospitalized with acute PE (Table 2).

**Figure 1:**
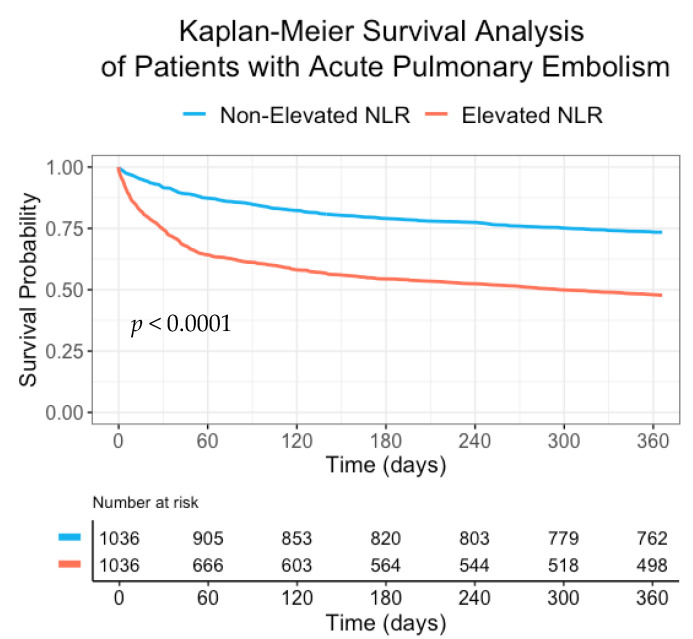
Kaplan–Meier survival curves for elevated and non-elevated NLR in patients hospitalized with acute pulmonary embolism.

In a sub-analysis of patients without a history of cancer (Supplementary Table S1) and patients with systolic blood pressure over 90 mmHg (Supplementary Table S2), similar trends were seen with an OR of 2.44 (95% CI 1.74–3.41) and 2.51 (95% CI 2.04–3.08), respectively. A Kaplan–Meier plot demonstrating survival probability in patients hospitalized with acute PE is shown in Figure 1.

**Figure 2:**
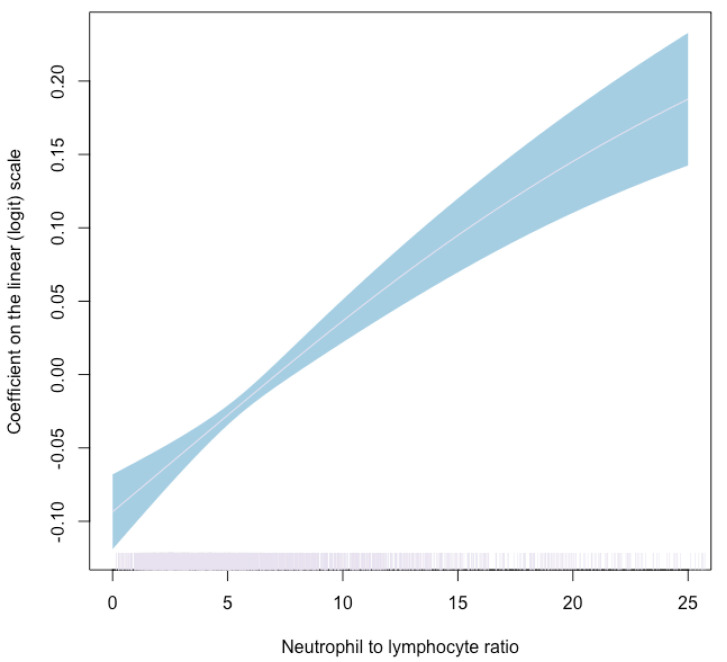
Results from a generalized additive model for the association between neutrophil to lymphocyte ratio (NLR) and the risk of 30-day mortality in patients hospitalized with acute pulmonary embolism. The coefficient of NLR is shown. The ribbon around the line shows the standard error. The vertical marks at the bottom show the distribution of NLR values in the sample.

The generalized additive model showed a positive, significant association between NLR levels and the risk of death within 30 days from the admission of patients diagnosed with acute PE (Figure 2). The strongest positive association was observed between NLR values of five and eight.

The median hospital length of stay in patients with an elevated NLR was higher (median of 5.84 days versus 3.09 days), and so was the in-hospital mortality rate (14.4% versus 3.4%) (Table 3).

## 4. Discussion

In this study, we investigated the prognostic value of NLR in predicting short and long-term mortality. We found a significant positive association between elevated values of NLR and mortality within 30 days and one year.

Our study demonstrated that elevated NLR, defined as a value above the median of 5.12, is associated with a higher 30-day and one-year mortality risk. Furthermore, results from a generalized additive model showed a significant positive association between NLR and the risk of death within 30 days from the admission of patients diagnosed with acute PE. The strongest positive association was observed for NLR values in the range of five to eight, emphasizing the fitness of our chosen cut-off value. A consistently positive slope is seen, illustrating the dose–response effect between NLR values and the 30-day mortality rate.

Elevated NLR values were shown to be associated with older age, lower systolic blood, higher pulse rate and lower oxygen saturation pressure at presentation and underlying comorbidities, including malignancy, heart failure, atrial fibrillation, hypertension, and chronic obstructive pulmonary disease. However, the association between elevated NLR and mortality within 30 days and 1 year persisted after adjusting for age, low blood pressure at presentation, and comorbidities. To further confirm these findings, we performed a sub-analysis of the patients without a known cancer diagnosis and hemodynamically stable patients at presentation (i.e., systolic blood pressure 90 mmHg or higher). The sub-analyses showed consistent findings, emphasizing the prognostic significance of NLR in the non-cancer population and hemodynamically stable patients. When investigating the secondary outcomes, we found that patients with elevated NLR have a longer hospital stay and higher rates of in-hospital mortality.

The relationship of NLR and PE prognosis has been previously reported [18,19,20,21,22,23,24]. However, previous studies were limited by relatively small sample sizes and broad exclusion criteria, preventing generalization. Moreover, most of these studies had a short follow-up period and therefore did not report long-term outcomes [18]. The present study consists of a large cohort size with 12 months follow-up, and apart from age (i.e., below 18 years old), no exclusion criteria were applied. In this study, we were also able to sub-analyze several populations of interest, such as hemodynamically stable patients (i.e., systolic blood pressure above 90 mmHg) and patients without a cancer background.

Risk stratification of patients diagnosed with acute PE is essential for determining the appropriate therapeutic management approach [6]. Although clinical, laboratory and radiologic factors may assist in weighing the likelihood of complications and mortality from PE, evaluating the prognosis of these patients remains challenging [6]. Several clinical scores for risk stratification are used, with the “Pulmonary Embolism Severity Index” (PESI) being the most validated [27,28]. NLR can be used as an addition to these tools, as a fast and simple measurement based on a routinely done laboratory test, requiring no additional cost.

## 5. Conclusions

To date, our study is the largest cohort investigating the short as well as the long-term prognostic role of NLR in patients hospitalized with acute PE. Elevated NLR in acute PE is associated with a worse short-term and long-term prognosis with a longer duration of hospitalization and higher in-hospital mortality risk. These findings, as well as the simplicity of their calculation, make the NLR a valuable prognostic marker and support its integration into clinical practice.

## 6. Study Limitations

This study has several limitations, the most important being its lack of information regarding management following diagnosis. This information was not available in our study. Therefore, the outcomes could be affected by differential therapy. Furthermore, imaging information with possible prognostic value such as thrombus size and location were not analyzed. As we did not compare patients with acute PE diagnosis to matched non-PE diagnosed patients, we cannot conclude whether NLR is a stronger prognostic marker in acute PE, rather than in alternate diagnoses.

## Figures and Tables

**Table 1:** Characteristics of patients at baseline.

	Non-Elevated NLR ^a^ (≤ 5.12)	Elevated NLR ^a^ (>5.12)	*p*-Value
*n*	1036	1036	
Sex [Male, *n* (%)]	440 (42.5)	442 (42.7)	0.965
Age (years) (median [IQR ^b^])	70.59 [56.79, 81.31]	76.01 [65.12, 84.61]	<0.001
Body mass index (kg/m^2^) (median [IQR ^b^])	26.60 [23.69, 30.87]	25.70 [22.90, 29.32]	<0.001
Temperature—Celsius (median [IQR ^b^])	36.80 [36.60, 37.00]	36.80 [36.60, 37.20]	<0.001
Pulse rate—beats per minute (median [IQR ^b^])	83.00 [74.00, 95.00]	89.00 [78.00, 102.00]	<0.001
Tachycardia [>100 beats per minute, *n* (%)]	172 (16.7)	283 (27.4)	<0.001
Systolic blood pressure—mmHg (median [IQR ^b^])	105.00 [92.00, 118.00]	97.00 [80.00, 111.00]	<0.001
Low systolic blood pressure [<90 mmHg, *n* (%)]	201 (19.4)	373 (36.1)	<0.001
Oxygen saturation (median [IQR ^b^])	96.00 [94.00, 98.00]	95.00 [92.00, 97.00]	<0.001
Low oxygen saturation [<90%, *n* (%)]	96 (9.4)	170 (16.7)	<0.001
Past PE ^c^ or DVT ^d^ (%)	124 (12.0)	143 (13.8)	0.238
Hypertension—*n* (%)	438 (42.3)	491 (47.4)	0.022
Ischemic heart disease—*n* (%)	125 (12.1)	150 (14.5)	0.120
Diabetes mellitus—*n* (%)	205 (19.8)	186 (18.0)	0.312
Cerebrovascular accident—*n* (%)	86 (8.3)	78 (7.5)	0.569
Heart failure—*n* (%)	44 (4.2)	77 (7.4)	0.003
Malignancy—*n* (%)	241 (23.3)	318 (30.7)	<0.001
Atrial fibrillation—*n* (%)	68 (6.6)	106 (10.2)	0.003
Chronic obstructive pulmonary disease—*n* (%)	63 (6.1)	127 (12.3)	<0.001
Chronic kidney disease—*n* (%)	72 (6.9)	88 (8.5)	0.217
Dyslipidemia—*n* (%)	279 (26.9)	297 (28.7)	0.404
Hemoglobin—g/L (median [IQR ^b^])	11.99 [10.50, 13.28]	11.40 [10.04, 12.96]	<0.001
Anemia ^e^ at presentation—*n* (%)	603 (59.0)	688 (67.5)	<0.001
Platelets—109/L (median [IQR ^b^])	219.00 [166.00, 284.00]	214.00 [162.00, 283.00]	0.767
International normalized ratio—*n* (median [IQR ^b^])	1.07 [0.99, 1.18]	1.11 [1.02, 1.23]	<0.001
Creatinine—µmol/L (median [IQR ^b^])	0.88 [0.72, 1.10]	0.94 [0.71, 1.29]	0.001
eGFR ^f^ below 60_mL/min/1.73m^2^_ at presentation—*n* (%)	293 (28.5)	435 (42.2)	<0.001
Troponin I—µg/L (median [IQR ^b^])	0.05 [0.03, 0.20]	0.05 [0.03, 0.19]	0.940
D-dimer—nmol/L (median [IQR ^b^])	1580.00 [700.00, 2923.00]	2062.00 [871.00, 4818.50]	<0.001

^a^ Neutrophil-to-Lymphocyte Ratio, ^b^ interquartile range, ^c^ pulmonary embolism, ^d^ deep vein thrombosis, ^e^ women hemoglobin <12 g/dL, men hemoglobin <13 g/dL ^f^ estimated glomerular filtration rate by CKD-EPI equation in adults (*Levey* et al., 2009).

**Table 2:** Adjusted odds ratios for the association between elevated NLR and the outcomes of 30-day mortality and one-year mortality in patients hospitalized with acute pulmonary embolism.

	aOR ^a^ for 30-Day Mortality (95% CI ^b^)	*p*-Value	aOR ^a^ for One-Year Mortality (95% CI ^b^)	*p*-Value
Elevated NLR ^c^ (>5.12)	2.82 (2.14, 3.7)	<0.001	2.51 (2.04, 3.08)	<0.001
Sex (males versus females)	0.95 (0.73, 1.24)	0.721	1.02 (0.83, 1.27)	0.835
Age	1.03 (1.02, 1.04)	<0.001	1.03 (1.02, 1.04)	<0.001
Atrial fibrillation	1.08 (0.72, 1.62)	0.719	1.17 (0.82, 1.69)	0.388
Ischemic heart disease	1.1 (0.77, 1.56)	0.614	1.13 (0.83, 1.54)	0.433
Heart failure	1.13 (0.7, 1.82)	0.628	1.03 (0.67, 1.59)	0.888
Chronic kidney disease	1.06 (0.69, 1.62)	0.796	1.06 (0.73, 1.55)	0.764
Diabetes mellitus	1.19 (0.87, 1.63)	0.278	1.41 (1.08, 1.84)	0.011
Cerebrovascular accident	1.03 (0.67, 1.6)	0.884	0.94 (0.65, 1.35)	0.735
Hypertension	1.04 (0.79, 1.37)	0.804	0.94 (0.75, 1.19)	0.626
Chronic obstructive pulmonary disease	0.87 (0.59, 1.31)	0.51	1.25 (0.89, 1.76)	0.19
Malignancy	1.72 (1.31, 2.25)	<0.001	3.4 (2.71, 4.27)	<0.001
Low systolic blood pressure	2.82 (2.19, 3.64)	<0.001	1.79 (1.43, 2.23)	<0.001

^a^ Odds-ratio, ^b^ confidence interval, ^c^ Neutrophil-to-Lymphocyte Ratio.

**Table 3:** Secondary outcomes of hospitalized patients with acute pulmonary embolism.

	Non-Elevated NLR ^a^ (≤5.12)	Elevated NLR ^a^ (>5.12)	*p*-Value
*n*	1036	1036	
Length of hospital stay (median [IQR ^b^])	3.09 [1.67, 7.45]	5.84 [2.81, 13.99]	<0.001
In-hospital mortality (%)	35 (3.4)	149 (14.4)	<0.001

^a^ Neutrophil-to-Lymphocyte Ratio, ^b^ interquartile range.

## Data Availability

Data are contained within the article or supplementary material.

## Supplementary Materials

The following are available online at https://www.mdpi.com/article/10.3390/jcm10184058/s1, Table S1: Adjusted odds ratio for the association between elevated NLR and the outcomes of 30-day mortality and one-year mortality in patients hospitalized with acute pulmonary embolism without history of cancer, Table S2: Adjusted odds ratio for the association between elevated NLR and the outcomes of 30-day mortality and one-year mortality in hemodynamically stable (i.e., systolic blood pressure over 90 mmHg) patients hospitalized with acute pulmonary embolism.
