# Supplementary material for: The Prognostic Role of Neutrophil-to-Lymphocyte Ratio in Patients Hospitalized with Acute Pulmonary Embolism
Source: J Clin Med. 2021 Sep 8;10(18):4058. doi: 10.3390/jcm10184058 (PMC8469500; doi:10.3390/jcm10184058)
Supplement: Supplementary file 1 [file jcm-10-04058-s001.zip › jcm-1315131-supplementary.pdf]

Journal of
Clinical Medicine

MDPI

Supplementary Materials

Supplementary Table S1. Adjusted odds ratio for the association between elevated NLR and the outcomes of 30-days mortality and one-year mortality in patients hospitalized with acute pulmonary embolism without history of cancer.

	Adjusted OR for 30-days mortality (95% CI)	Adjusted OR for one-year mortality (95% CI)
Elevated NLR	2.44 (1.74, 3.41)	2.57 (2, 3.31)
Sex (males vs. females)	1.06 (0.76, 1.47)	1.13 (0.87, 1.47)
Age	1.05 (1.04, 1.07)	1.04 (1.03, 1.05)
Atrial fibrillation	0.98 (0.61,1.56)	0.96 (0.64, 1.45)
Ischemic heart disease	1.03 (0.67, 1.58)	1.28 (0.89, 1.83)
Heart failure	1.51 (0.89, 2.58)	1.21 (0.75, 1.96)
Chronic kidney disease	1.02 (0.61, 1.69)	1.23 (0.8, 1.91)
Diabetes mellitus	1.08 (0.73, 1.6)	1.33 (0.97, 1.84)
Cerebrovascular accident	1.24 (0.75, 2.04)	1 (0.66, 1.52)
Hypertension	0.96 (0.68, 1.35)	0.94 (0.71, 1.24)
Chronic obstructive pulmonary disease	0.82 (0.51, 1.33)	1.23 (0.84, 1.82)
Low systolic blood pressure	3.41 (2.49, 4.68)	2.1 (1.61, 2.75)

Supplementary Table S2. Adjusted odds ratio for the association between elevated NLR and the outcomes of 30-days mortality and one-year mortality in hemodynamically stable (i.e., systolic blood pressure over 90mmHg) patients hospitalized with acute pulmonary embolism.

	Adjusted OR for 30-days mortality (95% CI)	Adjusted OR for one-year mortality (95% CI)
Elevated NLR	3.21 (2.31,4.46)	2.43 (1.91,3.08)
Sex (males vs. females)	0.57 (0.41,0.79)	0.88 (0.69,1.13)
Age	1.03 (1.02,1.04)	1.03 (1.02,1.04)
Atrial fibrillation	1.25 (0.74,2.11)	1.25 (0.81,1.92)
Ischemic heart disease	1.21 (0.78,1.89)	1.35 (0.94,1.92)
Heart failure	1.35 (0.74,2.47)	1.16 (0.7,1.94)
Chronic kidney disease	0.81 (0.46,1.44)	1.09 (0.7,1.7)
Diabetes mellitus	1.22 (0.82,1.79)	1.45 (1.07,1.98)
Cerebrovascular accident	0.92 (0.53,1.58)	0.75 (0.49,1.14)
Hypertension	0.9 (0.64,1.27)	0.92 (0.7,1.2)
Chronic obstructive pulmonary disease	0.89 (0.53,1.48)	1.14 (0.77,1.68)
Cancer	1.87 (1.34,2.59)	3.36 (2.59,4.37)
